# Strengthening intrapartum and immediate newborn care to reduce morbidity and mortality of preterm infants born in health facilities in Migori County, Kenya and Busoga Region, Uganda: a study protocol for a randomized controlled trial

**DOI:** 10.1186/s13063-018-2696-2

**Published:** 2018-06-05

**Authors:** Phelgona Otieno, Peter Waiswa, Elizabeth Butrick, Gertrude Namazzi, Kevin Achola, Nicole Santos, Ryan Keating, Felicia Lester, Dilys Walker

**Affiliations:** 1 Kenya Medical Research Institute, Nairobi, Kenya; 2 Department of Health Policy, Planning and Management, Makerere University School of Public Health, Kampala, Uganda; 3 Center of Excellence for Maternal, Newborn and Child Health, Makerere University, Kampala, Uganda; 4 Institute for Global Health Sciences, University of California, San Francisco, CA USA; 5 Department of Obstetrics, Gynecology, and Reproductive Sciences, University of California, San Francisco, CA USA

**Keywords:** Preterm birth, Neonatal mortality, Quality improvement, Simulation training, Kenya, Uganda

## Abstract

**Background:**

Preterm birth (birth before 37 weeks of gestation) and its complications are the leading contributors to neonatal and under-5 mortality. The majority of neonatal deaths in Kenya and Uganda occur during the intrapartum and immediate postnatal period. This paper describes our study protocol for implementing and evaluating a package of facility-based interventions to improve care during this critical window.

**Methods/design:**

This is a pair-matched, cluster randomized controlled trial across 20 facilities in Eastern Uganda and Western Kenya. The intervention facilities receive four components: (1) strengthening of routine data collection and data use activities; (2) implementation of the WHO Safe Childbirth Checklist modified for preterm birth; (3) PRONTO simulation training and mentoring to strengthen intrapartum and immediate newborn care; and (4) support of quality improvement teams. The control facilities receive both data strengthening and introduction of the modified checklist. The primary outcome for this study is 28-day mortality rate among preterm infants. The denominator will include all live births and fresh stillbirths weighing greater than 1000 g and less than 2500 g; all live births and fresh stillbirths weighing between 2501 and 3000 g with a documented gestational age less than 37 weeks.

**Discussion:**

The results of this study will inform interventions to improve personnel and facility capacity to respond to preterm labor and delivery, as well as care for the preterm infant.

**Trial registration:**

ClinicalTrials.gov, ID: NCT03112018. Registered on 13 April 2017.

**Electronic supplementary material:**

The online version of this article (10.1186/s13063-018-2696-2) contains supplementary material, which is available to authorized users.

## Background

Preterm birth, defined by the World Health Organization (WHO) as birth before 37 weeks’ gestational age (GA), and its subsequent health complications, are the leading cause of both neonatal mortality and under-5 child mortality [1]. Globally, an estimated one million newborns die each year due to complications of prematurity, and another 0.9 million preterm survivors suffer from mild to severe neurodevelopment impairments [2]. Thus, in order to further decrease child and neonatal mortality and morbidity, averting prematurity and helping preterm infants survive and thrive must be of highest priority.

In Kenya and Uganda, annual neonatal mortality rates have slowly decreased over the last decade, but remain high at 23 and 21 per 1000 live births, respectively [3]. Preterm birth rates are estimated to be 12.3% in Kenya and 13.6% in Uganda [4]. Both countries also have estimated high rates of stillbirth (per the definition of the WHO – a baby born with no signs of life at or after 28 weeks of gestation) with 21–23 deaths per 1000 total births [5]. Stillbirths are often not measured accurately and may disguise even higher rates of prematurity and/or neonatal mortality [6].

The largest burden of both overall neonatal, and, more specifically, preterm mortality, occurs within the first 24 h of life [7]. Similarly, a large proportion of stillbirths are intrapartum deaths, occurring less than 12 h before delivery and thus resulting in infants without any signs of maceration or skin deterioration (i.e., fresh stillbirths) [6]. Thus, the intrapartum and immediate postnatal periods represent critical windows of opportunity to improve neonatal outcomes in these settings. Estimates suggest that improved facility-based care during labor and birth and immediate newborn care can avert 0.8 million newborn deaths by 2025 [7]. These estimates reflect the potential of packages of interventions, rather than a single intervention, to make significant improvements in outcomes. However, many proven interventions are not widely used in many low- and middle-income countries (LMICs). Health system bottlenecks, like financial resources and workforce capacity limitations, have constrained systems’ abilities to deliver some interventions at scale [8].

Several approaches have been shown to improve the uptake of obstetric and neonatal evidence-based practices (EBPs) and interventions. First, the WHO developed the Safe Childbirth Checklist which includes 29 EBPs that focus on maternal and neonatal outcomes at four pause points – on admission to the facility, at the time of pushing (or before cesarean delivery), soon after birth (within 1 h) and at discharge. This checklist has been used in a variety of LMICs contexts, including India, Sri Lanka, and Namibia [9–11]. In Namibia, the authors reported an increase of EBPs from 68% to 95% over a 6-month period, as well as a reduction in perinatal mortality from 22 to 13.8 deaths/1000 deliveries [11]. A more recent report from the Better Birth Trial in India revealed that despite an increase in use of EBPs, outcomes did not improve [12], suggesting that the checklist alone may be insufficient in some contexts. Data from other countries’ implementation of the Safe Childbirth Checklist are still forthcoming, with the hypothesis that checklists improve uptake of EBPs by minimizing errors of omission or reminding the target audience to perform critical steps.

Second, simulation-based training is a technique used in many fields to immerse participants in a task or setting that simulates “real-world” contexts. In the obstetrics field, PRONTO International aims to optimize care during birth, through developing and implementing innovative in situ competency-based trainings that are grounded in highly realistic obstetric and neonatal simulation training. PRONTO trainings also emphasize teamwork and communication, and kind and respectful care, which contribute to empowering teams to identify system errors to catalyze change in their facilities. Increased EBPs related to appropriate management of the third stage of labor and neonatal care were observed in intervention clinics receiving PRONTO training as compared to controls in Mexico and Guatemala [13, 14].

Third, quality improvement (QI) is a strategy to optimize processes through testing of iterative changes. QI methods to improve quality of care have been described with various frameworks, such as the Plan-Do-Study-Act cycles [15]. In resource-limited settings, QI approaches have been used to improve effective scale-up of EBPs. For example, the Project Fives Alive! is a country-wide QI project in Ghana focused on the improved delivery of maternal and child health and nutritional interventions [16]. This project brings together QI teams at each facility that are responsible for the development and testing of “change ideas.” Members of facility teams form an Improvement Collaborative Network, giving the opportunity for sharing of failures, successes and ideas. Data from this study indicate that change activities were associated with increased postnatal attendance among underweight infants [17]. However, systematic reviews have demonstrated that QI studies have modest evidence and that more studies are needed to understand the necessary and sufficient elements of QI strategies [18].

Lastly, in order to understand the impact of any interventions on maternal and neonatal outcomes, robust measurement of data must be prioritized. The Every Newborn Action Plan (ENAP) published 10 core indicators that should be tracked in order to improve quality of care for mothers and newborns [19]. This ENAP roadmap underscores the importance of standardizing indicator definitions and strengthening routine health information systems. In the field of prematurity, in particular, data strengthening around accurate GA using available resources and systems has been identified as a key issue. For example, in a study examining the use of antenatal corticosteroids in LMICs, birthweight of below the 5th percentile was used as a proxy measure for preterm birth because GA recording was considered of insufficient accuracy [20].

To our knowledge, no published studies have examined the impact of a facility-based intervention package focused on improving uptake of obstetric and neonatal EBPs in order to address preterm mortality. The East Africa Preterm Birth Initiative (PTBi-EA) hypothesizes that a facility-based intervention package administered during the intrapartum and immediate postnatal period will decrease the neonatal mortality rate among preterm neonates. Our package comprises four components: (1) strengthening of routine data collection and data use activities, including regular data quality assessments (DQAs); (2) implementation of the WHO Safe Childbirth Checklist modified for preterm birth; (3) PRONTO provider training and mentoring on key EBPs to strengthen intrapartum and immediate newborn care; and (4) support of QI teams. We believe that these interventions, in combination, will improve awareness and practice of EBPs; teamwork, communication, and respectful maternity care; and use of data for decision-making. We anticipate that results from this study will inform how interventions used in combination can improve personnel and facility capability and readiness to respond to preterm labor and delivery, as well as care for the preterm infant.

## Methods

### Trial design

**Fig. 1:**
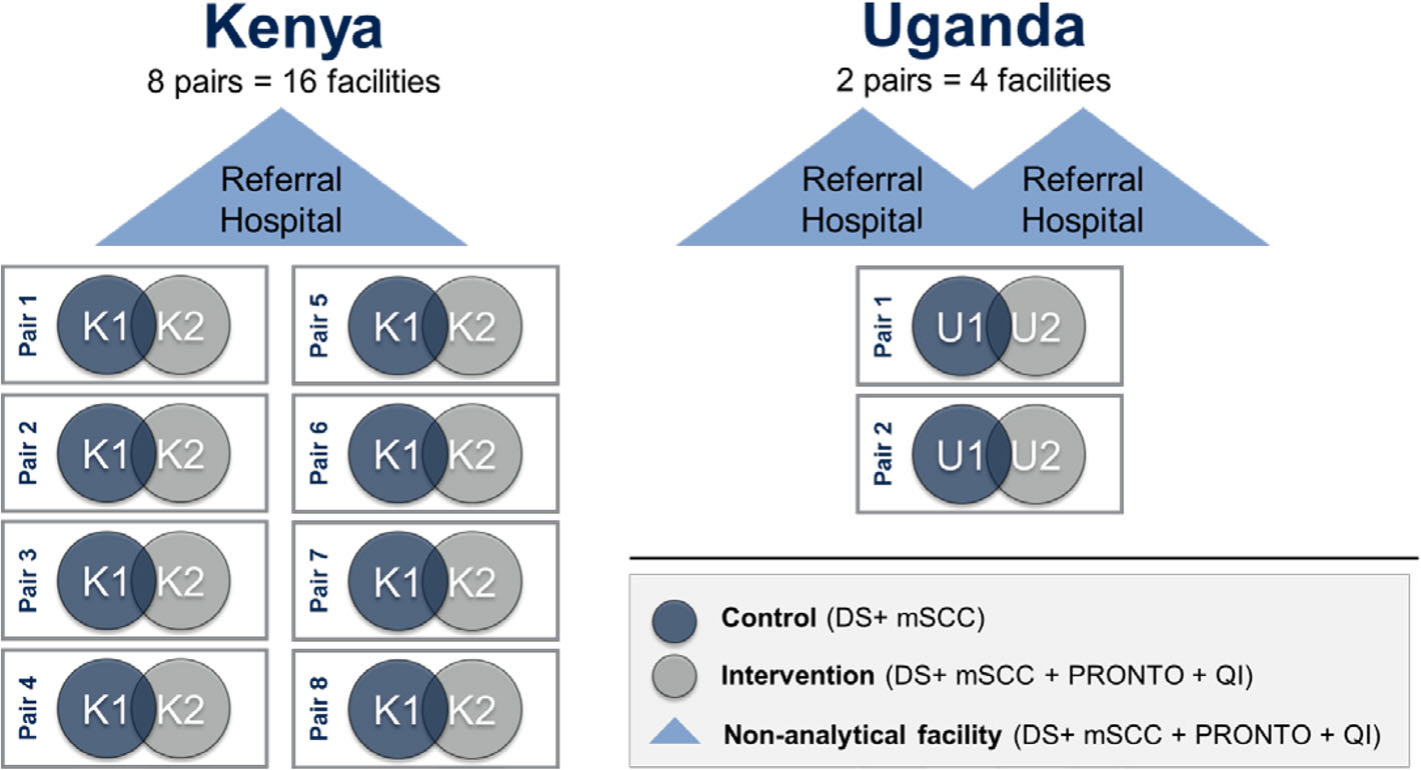
Schematic of the study design

This study is a pair-matched, cluster randomized controlled trial (CRCT) among 20 public sector health facilities in the Busoga Region of Uganda (four facilities) and in Migori County, Kenya (16 facilities, including 14 public facilities and two not-for-profit missionary hospitals). The full intervention package (data strengthening (DS), modified Safe Childbirth Checklist (mSCC), PRONTO provider training, and quality improvement (QI)) will be introduced to 10 facilities (intervention arm); the remaining 10 facilities will receive DS + mSCC (control arm) (Fig. 1). All facilities will begin with DS + mSCC intervention components in order to capture preliminary data for baseline and facility matching, as well as to standardize definitions of key indicators related to GA and newborn outcomes. Roll-out of mSCC and support will differ between the control and intervention sites, in that the latter will receive additional mSCC mentorship and support through synergies with PRONTO and QI. The intervention package will be delivered at the facility level, while outcomes will be measured at both an individual and facility level.

In addition to the 10 pairs of matched facilities, three referral hospitals to which the respective sub-county or district hospitals send their high-risk deliveries will receive the full intervention package. While these three referral hospitals are not included in the randomization scheme, cases referred in from any one of the 20 facilities will be included in the primary outcome analysis.

### Setting and site selection

The study regions of Migori County, Kenya and Busoga Region, Uganda were selected based on in-country stakeholder input. Prematurity burden and presence of synergistic parallel maternal/newborn research or implementation studies were considered. Health facilities were asked to participate in this study by in-country partners. Formal approval from facility leadership was obtained before any activities commenced. Given that facilities were not selected from a target population of hospitals, the intervention effects should be interpreted as impact evaluation of the intervention package implemented at the said facilities. A complete list of all facilities can be found at the clinical trial registration (ClinicalTrials.gov, ID: NCT03112018).

The Busoga Region of Eastern Uganda contains approximately three million people, or 10% of Uganda’s population, with over 80% of residents living on less than US$1 per day [21]. The estimated preterm birth rate for Uganda is 13.57% and the neonatal mortality rate is 21 per 1000 live births [3, 4]. Our selected six health facilities include approximately 22,000 deliveries per year, with 9000 deliveries from the four hospitals pair-matched in this study. Migori County, located in southwestern Kenya, has a population of approximately 917,170, wherein 43% of the population lives below the poverty line [21]. The estimated preterm birth rate for Kenya is 12.30% and the neonatal mortality rate is 23 per 1000 live births [3, 4]. Our 17 selected Migori County health facilities include approximately 10,000 deliveries per year, with 7500 deliveries at pair-matched sites.

### Study population

In all facilities, women accessing delivery care services who are admitted for labor will be eligible for this study. Anonymized data on all deliveries will be extracted from maternity registers. For follow-up, mothers of newborns who are discharged alive and born less than 2500 g or between 2501 g and 3000 g while also being identified as less than 37 weeks by recorded GA in the maternity register are being asked to participate in this study and approached for consent for follow-up for 28-day outcome. These inclusion parameters were selected based on baseline data showing a poor correlation between birthweight and reported GA in the maternity register. Women or newborns from enrolled sites who are referred to one of the three referral facilities will remain in the study. Their outcomes will be allocated to the facility to which the woman first presented.

Healthcare workers providing labor and delivery and immediate newborn care services at referral, control, and intervention facilities will participate in DS initial and refresher workshops, DQAs, as well as initial instruction and minimal refreshers or reorientation on use of the mSCC. Intervention sites will have ongoing support for mSCC utilization, in addition to the other reinforcing intervention components, PRONTO and QI. Healthcare providers in intervention facilities who provide consent for PRONTO simulation trainings will also be enrolled as study participants to ascertain changes in knowledge and practices. Facility staff in the intervention facilities will be organized into QI teams to develop and implement QI programs.

### Intervention package components

Ten intervention sites and the three referral-level hospitals will receive an intervention package comprising DS + mSCC + PRONTO + QI, while the remaining 10 control facilities will receive DS + mSCC. Each intervention component is described in detail below. The intervention package is designed to strengthen and reinforce EBPs, as well as improve teamwork, communication, respectful maternity care and data use. All study activities consist of known interventions or strategies. There are no experimental interventions that would directly impact patient safety.

#### Data strengthening (DS)

Improvements in measurement and data use in the study sites are critical to establishing baseline measures and achieving and demonstrating reductions in the burden of preterm birth. Therefore, we will begin our study by strengthening existing data collection processes in health facilities, introducing standard tools to improve GA assessment, and reviewing standardization of indicators based on national guidelines. We will also work with in-country stakeholders to develop and iterate a Data Dashboard to improve data use and dissemination of our study data (Table 1).

**Table 1:** Components of data strengthening

Data strengthening component	Description
Reinforcing current status of data systems and indicators	We will provide technical support to standardize definitions of indicators currently being collected, improve adherence to national guidelines on labor and delivery documentation of registers, improve quality of reporting, and strengthen existing data quality assurance and data use processes. Follow-up assessments to gauge improvements in facility systems including data quality assessments (DQAs) will be conducted at regular intervals.
Refining standardized gestational age measurement	We will strengthen the use of last menstrual period (LMP) with pregnancy wheels to accurately calculate gestational age (GA). We will reinforce more accurate measures of birthweight by providing training and assessing calibration of facility scales at regular intervals.
Developing a Data Dashboard	To improve data use and dissemination of routine data, we will create a synchronized online Data Dashboard repository system that is adaptable for local providers, health officials, and national policymakers. This tool will be based on discussions among various PTBi stakeholders to better understand and respond to data needs, and is also integrated in QI and project monitoring and evaluation.

DS initial training will include review of these components with facility clinical leadership, health records officers and district staff, followed by site trainings with maternity nurses and health record staff. Refresher DS trainings will be offered as needed during the course of the study. Intermittent DQAs will be implemented every 6–12 months to collect data on specific DHIS-related indicators to assess gaps between reported and actual indicators (e.g., errors in transcription) across all control and intervention facilities. This process will also help identify barriers in the reporting processes and flow.

#### Modified Safe Childbirth Checklist

Each country team will modify the WHO Safe Childbirth Checklist in order to adhere to their national guidelines. It will be adapted to the local setting and modified to emphasize identification of preterm labor and care of the preterm infant. Specifically, we will incorporate additional elements focused on GA assessment and documentation, prematurity-related intrapartum/immediate postnatal care practices (e.g., use of magnesium sulfate, antenatal corticosteroids, immediate skin to skin, etc.). We will also include an additional pause point at initial presentation or triage (i.e., before a woman is admitted for labor), as well as prompts focused on ascertaining additional maternal demographic information, clinical risk factors and history.

Each country’s mSCC will be piloted in order to optimize content and roll-out. The mSCC will be introduced during initial DS activities at all study sites. It is intended to serve as both a decision aid for providers of key EBPs, as well as a data source for the study. An mSCC will be included in the maternity inpatient record for each woman in all control and intervention facilities.

After piloting, study data staff will review all maternity charts of cases eligible for follow-up each month and abstract a selected number of essential data variables. Study personnel will also monitor mSCC completeness and uptake by each of the five pause points either by convenience or purposive sampling. These data will be displayed on the Data Dashboard quarterly, and will allow the study teams to tailor the mSCC approach depending on uptake and use.

#### PRONTO simulation-based training

The Kenya-Uganda unified PRONTO emergency obstetric and neonatal care simulation training will emphasize the identification, triage, and management of preterm labor and birth with a curriculum specifically adapted for this context. It will include strengthening preterm labor identification with more accurate GA assessments, intrapartum care, and immediate management of fragile infants. The training also emphasizes identification and management of preeclampsia, chorioamnionitis, and other conditions related to preterm birth. The mSCC will be integrated into all PRONTO clinical activities to provide facility staff with continued opportunities to reinforce its use. Any change ideas that arise from these PRONTO activities will be integrated into QI efforts.

Selection of PRONTO mentors/trainers will be conducted in each country, with an initial pool of up to 15 candidates from which we will select the 5–10 highest performing trainers. Due to the overlapping clinical and curricular content between the two countries, refreshers will be conducted as joint facilitator training for the Kenyan mentors and Ugandan trainers. However, the training mode of delivery will vary between Kenya and Uganda. Kenya will utilize an in situ mentoring program whereby each intervention facility will receive high-intensity/4-day per week mentorship and a pair of mentors will rotate among intervention sites during the study duration. They will spend a combined total of 9–12 weeks at each intervention facility over the study duration and visits will include bedside mentoring, video-recorded, in situ simulations, knowledge reviews, skills stations, teamwork activities, and mSCC support. In Uganda, a high-intensity/shorter modular strategy will be used. A modular-based training program will be paired with 2-day-long in-situ simulation refresher and training visits. Modules and refresher/training visits will be spread out during the study period, and will similarly amount to approximately 6 weeks of mentorship. Thus, while the mode of delivery for training in each country will be different, provider teams in each country will receive approximately 56–58 h of PRONTO-based instruction using the same curriculum.

#### Quality improvement (QI) cycles

Each facility in the intervention arm will have a designated QI team comprising facility leadership, nurses, and health record staff (five to seven people). If teams have been trained previously through other QI efforts, we will revitalize and support these ongoing efforts in intervention sites. Otherwise, we will offer foundational training in QI methods. These teams will carry out Plan-Do-Study-Act cycles which include identification of a problem or bottleneck in the facility, implementation of solutions, tracking of the outcomes of the changes, and implementation or adjustment based on the results.

QI teams across facilities (known collectively as the QI collaborative) will also participate in a learning session every 3 to 6 months to discuss core learnings and QI indicators. QI indicators will be chosen by each country and will focus on EBPs expected to result in decreased neonatal mortality among preterm infants, such as uptake of Kangaroo Mother Care, antenatal corticosteroid provision, and breastfeeding. At these learning sessions, QI teams across facilities will be able to share progress on QI indicators, lessons learned, and best practices.

Elements of the package, namely PRONTO, the Data Dashboard and the mSCC, will be integrated with QI efforts. First, the Data Dashboard will help generate specific visual data to inform facility teams on progress and remaining performance gaps with respect to QI indicators and selected EBPs. Second, areas of possible improvement that arise through PRONTO mentorship and simulation will be shared with QI teams. Lastly, the mSCC may serve as a data source to document QI indicators.

### Intervention roll-out

The approach for roll-out of the intervention package will be as follows:

- *Baseline*: Across all selected facilities, preliminary DS with initial introduction of the mSCC will allow us to conduct a baseline survey to measure incidence of preterm births and preterm mortality in all facilities (at least 2 months). Baseline data will also be collected on key variables that are predictive/correlated with the primary outcome of mortality rate among preterm infants, including but not limited to number of deliveries, number of low birthweight babies, number of neonatal deaths, number of stillbirths, presence of a newborn corner, personnel capacity for intrapartum/postnatal care, etc.
- *Step 1*: Facilities will be pair-matched using key factors correlated with outcomes. Specifically, sites will be pair-matched based on variables collected during baseline that are predictive or correlated with the primary outcome of mortality rate among preterm neonates (i.e., delivery volume, number, and type of providers, facility-based neonatal mortality rate).
- *Step 2*: Within each matched pair, one facility will be randomized to the intervention arm and the other will be randomized to the control arm. Intervention facilities will receive PRONTO and QI interventions in addition to DS and mSCC. Randomization and allocation will be done using a computerized random number generator by the statistical team with no contact or direct interest in any specific facilities. As this is a cluster trial, there will be no blinding or allocation concealment.
- *Step 3*: Upon accruing sample size, the control arm facilities will be assigned to the full intervention, adding PRONTO training and QI cycles.

**Fig. 2:**
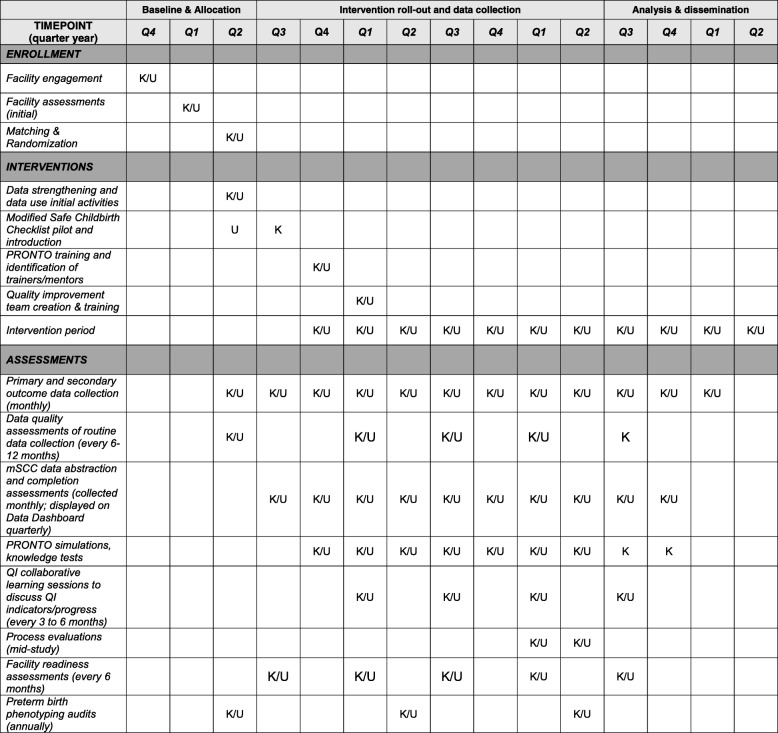
Standard Protocol Items: Recommendations for Interventional Trials (SPIRIT) Figure

Figure 2 depicts the schedule of enrollment, interventions, and assessments to be conducted.

### Outcomes

The primary outcome for this study is 28-day mortality rate among preterm infants. For this primary analysis, the denominator will include:

- All live births weighing greater than 1000 g and less than 2500 g
- All live births weighing between 2501 g and 3000 g with a documented GA of less than 37 weeks
- All fresh stillbirths weighing greater than 1000 g and less than 2500 g
- All fresh stillbirths weighing between 2501 g and 3000 g with a documented GA of less than 37 weeks

This outcome will be measured by comparing mortality rates of preterm infants at 28 days after delivery across the intervention and control arms to determine the effect of the package of facility-level interventions. The upper limit of 3000 g (which is coupled to a documented GA of less than 37 weeks) was agreed upon based on INTERGROWTH-21st standards. At this weight, we assume that we will capture 90% and 97% of < 34-week infants and 60% and 70% of < 37-week infants, for female and male children, respectively [22]. The lower limit of inclusion for the primary analysis was set at 1000 g to align with the *International Classification of Diseases 10th revision* (ICD 10) definition of late fetal death (i.e., birthweight of > 1000 g or > 28 weeks’ gestation) [6]. However, data on infants with signs of life born weighing < 1000 g will be followed for secondary data analyses, including outcomes at 28 days of life. Selected secondary outcomes are listed in Table 2.We include additional information as to how they will be measured and how often during the study duration.

**Table 2:** Select secondary outcomes

Secondary outcome	How variable will be measured	Time frame
Data quality of key indicators in facility-based registers (includes GA, facility discharge status, preterm birth incidence)	GA-birthweight concurrence, DQAs and mSCC data review or QI indicators	Baseline through study completion, an average of 18 months; at least quarterly
Pre-discharge mortality among all infants > 1000 g	Clinical record review at facility	Baseline and through study completion, an average of 18 months
Facility-based maternal mortality	Clinical record review at facility	Baseline and through study completion, an average of 18 months
Perinatal mortality (fresh stillbirths and deaths within 7 days) among eligible preterm infants (includes pre-discharge mortality)	Parental report at 28 days and clinical record review at facility	Baseline and through study completion, an average of 18 months
Mortality among preterm infants and those born alive between 500 g and 999 g at birth (include pre-discharge mortality and 28-day mortality)	Parental report at 28 days and clinical record review at facility at time of first contact	Baseline and through study completion, an average of 18 months
Average number of EBPs or Ministry of Health management guidelines demonstrated in simulated case videos and live birth observations	Measured in PRONTO simulation videos, observed live births and/or mSCC. To be complemented by pre-post knowledge tests and facility assessments	Baseline and through study completion, an average of 18 months. PRONTO administered at pre-determined time points
Knowledge improvement of EBPs	PRONTO pre-post knowledge test scores	PRONTO administered at pre-determined time points
Proportion of eligible cases receiving EBPs reported by QI teams (QI indicators include Kangaroo Care, antenatal corticosteroid provision, and breastfeeding)	QI indicators reported at learning sessions	Baseline and through study completion, an average of 18 months; QI learning sessions held every 3–6 months
Facility readiness to handle delivery and newborn complications	Measured by a facility assessment tool	Bi-annually over the study period
Understanding of contextual factors influence implementation of strengthening maternal and newborn care interventions	Process evaluation that incorporates document review, surveys and qualitative interviews and focus groups	To be conducted mid-study
Prevalence of preterm birth phenotypes in the study sites	Retrospective and/or prospective chart review	Baseline and through study completion; at least annually in select sites

*GA* gestational age, *DQA* data quality assessment, *EBP* evidence-based practice, *mSCC* Modified Safe Childbirth Checklist, *QI* quality improvement

### Data collection/quality control

For the primary outcome, existing facility-based registers will be used as primary data sources. Data entry into registries is conducted by facility care providers, as part of existing routine data collection. Information in these registries will then be extracted by study personnel. Study staff will visit each facility at least once per month to collect routine facility data from register reviews. All data will be uploaded via Open Data Kit via tablet or laptop.

For preterm babies discharged alive from hospital, mothers will be consented to be followed up by phone at 28 days following delivery. Consent of eligible mothers and their newborns will take place prior to discharge or referral. Contact information will be derived from consent forms, and from the mSCC as needed. Outcomes will be determined by targeted follow-up of study participants via phone call. Where phone calls are insufficient to trace mothers, the Kenya team will engage with community health volunteers and Uganda will employ community outreach nurses to seek out mothers.

#### Data strengthening

Study personnel in each country will collect data from maternity registers on a monthly basis. In addition to reviewing the quality of records, they will also generate summary reports for data sharing among the research team and facility leads.

Routine indicators and process indicators for QI cycles will be collected and displayed on a Data Dashboard accessible to study staff, intervention and referral health facility staff, and county health authorities. While these Data Dashboard displays will be customized to different audiences, all of the data included will be aggregate, and no individual data will be displayed.

#### Modified Safe Childbirth Checklist (mSCC)

The mSCC will be distributed to facilities and fixed into the patient charts in readiness for use by the healthcare providers. Staff will be adequately trained on the use of the checklist and regular reinforcement conducted as scheduled at intervention sites. Study staff will review all maternity charts for newborns who meet our eligibility criteria. Each month, they will abstract key data from eligible admissions from the mSCC in order to compile coverage indicators for key interventions and EBPs. Uptake and completeness of the mSCC will also be determined.

#### PRONTO simulation-based training

To measure changes in knowledge through training and mentoring, we will conduct evaluations in the form of pre- and post-knowledge tests of PRONTO-trained midwives, nurses, and physicians before and after each training session and periodically during mentoring visits. These evaluations will be adapted to the local context based on previously developed knowledge assessment tools used by PRONTO. To evaluate the impact of PRONTO’s on-site simulation training program, we will collect video-recordings of simulated birth scenarios and debriefs conducted in participating hospitals led by PRONTO-trained mentors/trainers. These videos will be coded using Studiocode™ software to create scores based on how often EBPs are practiced in simulation and if this changes over time.

#### Quality improvement (QI) cycles

We will track process indicators of these QI cycles, such as number of projects started, number of goals reached, and amount of change detected by the QI team in studying their implementation. We will implement a QI documentation journal for the sites. QI teams will also track key EBPs, such as Kangaroo Care uptake, and track their progress against it.

### Data management

Data from registries will be collected using a secure database via the Open Data Kit data entry platform and hosted on a secure server. Data will be reviewed for accuracy and completeness by a data manager before entry, and the data entry system will include automated range and logic checks to identify any data entry mistakes before they are saved. All devices used for data entry (laptops or tablets) will be encrypted and password protected.

The research team and stakeholders will have access to aggregate data across facilities through the Data Dashboard. For example, each facility will have access to their own data including 28-day outcome, but stratified data by control and intervention data will not be shared. Moreover, only the study biostatistician and core team will have access to the unblinded dataset prior to study completion.

### Sample size and power calculations

Our primary analysis will combine data across our selected facilities in Kenya and Uganda. Since we will exclude the referral hospitals from our primary analysis, the project takes place in 20 facilities with an expected volume of 46,000 deliveries over 24 months. In an initial calculation, prior to baseline data collection, we assumed an average preterm birth rate of 12% and expected to see about 5500 preterm deliveries within this period. We assumed a 25% loss-to-follow-up rate for eligible cases. This yields at least 200 projected preterm deliveries per facility with known 28-day mortality outcome.

Detectable effect sizes were estimated by standard *t* test procedures adjusted to account for the design effect due to clustering of outcomes within facilities to attain 80% power while maintaining type I error at 5%. At a 25% 2-year cumulative incidence of 28-day mortality across both countries in the absence of the intervention, this would allow us to detect a 25% reduction in cumulative incidence if the between-cluster outcome coefficient of variation is 0.2 or below. If this coefficient increases to 0.3, we would be powered to detect a 30% reduction. If it increases to 0.4, we would be powered to detect a 40% reduction.

### Analyses plans

For the primary outcome, the analysis will contrast mortality at 28 days among preterm infants between the intervention and control groups. This will be performed using hierarchical, targeted maximum-likelihood estimation which accounts for within-cluster correlation by controlling for cluster-level covariates [23]. The baseline covariates we will measure for each facility include delivery volume, baseline neonatal mortality rate among preterm infants, preterm birth rate, and country. Additionally, targeted maximum-likelihood estimation will allow us to incorporate individual-level baseline covariate information (such as date of presentation, maternal age, parity/gravidity, HIV status, maternal and fetal complications at presentation, last menstrual period (LMP), infant birthweight, final diagnosis of newborn) in order to improve precision. We will directly incorporate knowledge of the pair-matched randomization scheme into estimation by making the targeting stage of this procedure a function of this known assignment mechanism. Primary outcome data analysis will be conducted in collaboration among in-country partners and UCSF.

Secondary analyses will also be performed, in some cases contrasting secondary outcomes between the intervention and control groups, and in others contrasting these outcomes pre and post intervention within the intervention groups. Secondary analyses will primarily be descriptive, comparing means or proportions between groups, trends over time, composite scores as appropriate for each measure. Both primary and secondary analyses will be conducted in R or Stata. Process data including qualitative interviews or reports will be analyzed by hand or in Atlas ti, identifying and grouping themes that emerge.

Additional analyses will be conducted for each intervention component. For example, PRONTO-related knowledge will be assessed against the standard guidelines for management. Simulation and debrief videos will be analyzed using Studiocode™ software which enables the systematic coding of videos to measure of changes in use of EBPs, as well as teamwork and communication techniques. Video analysis of simulation and debrief results will be shared with participants in the form of structured feedback to PRONTO mentors on their simulation and debriefing facilitation skills.

### Result dissemination

In accordance with the Bill & Melinda Gates Foundation open-access policy, we will publish in open-access journals. The final trial dataset will be made publicly available after study completion once all datasets are cleaned and initial results are reported. We plan to disseminate evaluation findings to both internal and external stakeholders, including facility staff implementers, Ministries of Health policymakers, and the Bill & Melinda Gates Foundation.

### Ethical considerations

The intervention package is designed to strengthen and reinforce best EBPs, and all study activities consist of known interventions or strategies. There are no experimental interventions that would directly impact patient safety. Should any adverse events be reported, these will be immediately reported to study leadership and ethics committees.

#### Approvals

This proposal was submitted to KEMRI, Makerere University School of Public Health, and UCSF Scientific and Ethical Research Bodies for scientific and ethical approval before study initiation.

#### Consent procedures

This is an implementation science study and intervention components will be applied to facilities rather than individuals. In most cases, there will be no direct contact with the participants except for the 28-day follow-up. For the primary outcome, risks to participants will be minimized by the fact that facility registers and medical records will be used as the primary source of information and no identifiable information will be collected or used. Data abstraction from registers, medical records and the mSCC will be conducted in a private, confidential area of each facility. However, for 28-day follow-up among eligible newborns, mothers will be asked to provide written consent prior to discharge. No incentives will be provided, and women can opt out from participation.

For providers in the intervention arm, both PRONTO mentors and mentees will be asked for written consent authorizing the use of knowledge tests and video data for analysis. While QI indicators and a change in direction of performance would be noted over the course of the study, QI team members and facility staff will not be asked for consent as no identifying individual data will be collected.

## Discussion

This study focuses on facility-based care of mothers and infants during the intrapartum and immediate newborn period. Across Kenya and Uganda, as well as each of the study facilities, the intervention components will be tailored by in-country stakeholders. For example, the mSCC and PRONTO curriculum will be adapted to ensure adherence to national guidelines. If ongoing QI is already in place at our selected intervention facilities, QI teams and strategies will synergize with these existing efforts or program. This will ensure alignment of the trial to current policies and practices.

**Fig. 3:**
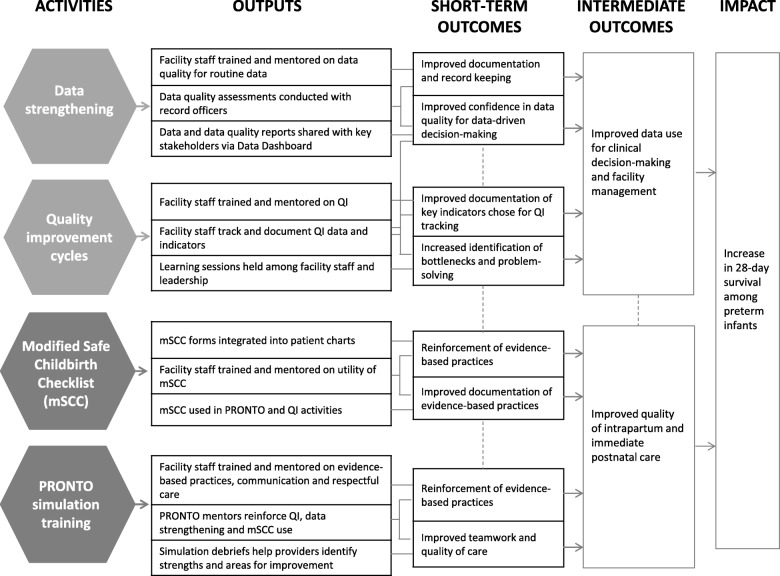
Logic model for study interventions

The trial, as designed, will assess a package of interventions rather than a single intervention. This approach reflects a consensus in the field that multiple interventions are needed, but creates challenges for researchers accustomed to being able to attribute effects more granularly to specific interventions. We believe that each intervention component reinforces each other to create a synergistic package that, when implemented together, will allow facility providers to improve EBPs and their appropriate documentation (Fig. 3). The interventions target different types of facility staff and cadres of providers, demonstrating that shared and cooperative strategies are needed to address quality of care.

### Challenges and opportunities

Several challenges will need to be overcome in order to ensure the success of this study. First, GA accuracy remains to be a major obstacle in these settings. Lack of early ultrasound, coupled with late antenatal care-seeking practices and inherent challenges with using LMP, makes GA difficult to robustly capture. Second, using routine data sources may uncover challenges with data quality and completeness. While data strengthening is a foundational intervention across all of our sites, staff turnover, industrial strikes, burnout, and other contextual factors may impact data. Third, although this is a package of interventions, each of the component parts has its unique strengths and challenges. Uptake and acceptability of the individual components may vary. Lastly, our trial focuses on improving facility-based care during the intrapartum and immediate newborn period; however, our primary outcome is 28-day mortality among preterm infants (denominator defined as the sum of live born infants weighing greater than 1000 g and less than 2500 g or GA of less than 37 weeks and weight less than 3000 g plus fresh stillbirths with the same weight and GA criteria). The ability to monitor post-discharge health or activities will not be possible within the scope of this project.

Several research opportunities arise within this trial. First, as preterm birth is often described as a syndrome [24], this study will allow for nested studies examining maternal, fetal, and placental risk factors that contribute to preterm birth. As such, preterm birth phenotyping studies using the framework described by Barros et al. (2012) will be nested at some of our sites [25]. Second, as GA assessment will prove to be a challenge in many of our sites, this poses a unique opportunity to test other measures to more accurately estimate GA. This may include, for example, studies focused on discovery of biomarkers and/or comparison of various anthropometric measures.

This study describes a single CRCT that spans regions in Western Kenya and Eastern Uganda. The interventions implemented will need to be tailored and adapted to the local context and national guidelines. It will also be important to document any overlapping or contributing factors of other ongoing or newly introduced maternal or newborn health initiatives that may impact our study results, such as other QI initiatives or training programs. Nonetheless, this is a great opportunity to demonstrate both the feasibility and challenges associated with adapting interventions under a shared research study and outcome measure.

## Trial status

This trial has completed planning and began enrollment in October 2016. Study enrollment is ongoing with expected completion by October 2018.

### Trial registration

Preterm Birth Initiative Kenya/Uganda Protocol dated: 19 December 2016; ClinicalTrials.gov, ID: NCT03112018.

## Additional file

Additional file 1: Standard Protocol Items: Recommendations for Interventional Trials (SPIRIT) Checklist. (DOC 123 kb)

## Abbreviations

DQA: Data quality assessment
DS: Data strengthening
EBP: Evidence-based practice
ENAP: Every Newborn Action Plan
GA: Gestational age
LMIC: Low- and middle-income country(ies)
LMP: Last menstrual period
mSCC: Modified Safe Childbirth Checklist
PTBi-EA: East Africa Preterm Birth Initiative
QI: Quality improvement
WHO: World Health Organization
